# The clinical back pain courses described by information available in Danish central registries

**DOI:** 10.1186/s12913-021-07409-w

**Published:** 2022-01-06

**Authors:** Maria Iachina, Olav S. Garvik, Pernille S. Ljungdalh, Mette Wod, Berit Schiøttz-Christensen

**Affiliations:** 1 Center for Clinical Epidemiology, Odense University Hospital, and Research Unit of Clinical Epidemiology, University of Southern Denmark, Sdr. Boulevard 29 entrance 219, 1. Floor, DK-5000 Odense C, Denmark; 2 Medical Research, Spine Centre of Southern Denmark, University hospital of Southern Denmark, Lillebaelt, Østre Hougvej 55, 5500, Middelfart, Denmark; 3 Department of Regional Health Research, University of Southern Denmark, J.B. Winsløws Vej 19, 3, 5000 Odense C, Denmark

**Keywords:** Back pain, Registries, Hospital departments

## Abstract

**Background:**

Patients with back pain are often in contact with 2–4 hospital departments when receiving a back pain diagnosis and treatment. This complicates the entire clinical course description. There is, currently, no model that describes the course across departments for patients with back pain. This study aims to construct an interdisciplinary clinical course using the central register’s information.

**Methods:**

All patients with back pain referred for diagnosis and treatment at the Spine Center of Southern Denmark from 1 January 2011 until 31 December 2017 were included. By means of information available in central registers, we described the interdisciplinary clinical course for the individual patient, including information on all contacts at different departments, and proposed three different models to define the index and final date. The index date was defined as the first visit without a previous contact to the Spine Center for 6 months for model I, 1 year for model II, and 2 years for model III. The final date was defined as the last visit without following contacts for 6 months, 1 year, and 2 years, respectively, for models I, II, and III.

**Results:**

A total of 69,564 patients (male: *n* = 30,976) with back pain diagnosis were identified. The three models all leave the information on the entire course at the hospital. In model I (64,757 clinical back pain courses), the time span to a possible previous clinical course is too short to secure the start of a new course (14% had two or more). With at least 1 year between a possible previous contact, model II (60,914 courses) fits the everyday clinical practice (9% had two or more clinical back pain courses). In model III (60,173 courses) it seems that two independent courses might be connected in the same course as only 5% had two or more clinical back pain courses.

**Conclusions:**

Despite contact with different departments, the clinical course for back pain patients can be described by information from the central registers. A one-year time interval fits best the clinicians’ everyday observations.

## Background

In 2010 the Danish National Health profile showed that about 20% of the Danish population reported low back pain in the preceding 14 days [1]. The latest report on clinical quality in Denmark showed that up to 100,000 patients with back disorders are examined in hospitals every year. Approximately 65,000 individuals of those (14%) undergo surgery. Every year approximately 30,000 develop chronic back disorders [2]. An estimate shows that patients with back pain represent 15.5% of all consultations at general practitioners and that 53.2% of all visits to chiropractors and physiotherapists in Denmark are due to back pain [1–3]. Despite medical advances and extensive occupational safety measures, the influence back pain has on the patients continues to increase [4–6]. Back disorders and musculoskeletal disorders, in general, present a significant public health problem, limiting productivity and imposing a substantial socioeconomic burden on the society [7–10].

Patients with persistent disabling back pain may need a referral to specialized hospitals for diagnostics and treatment procedures such as surgery. Even though approximately 25% of the patients seen in the primary sector are referred to specialized hospital departments every year, due to back pain, we know very little about the clinical course and these patients’ characteristics (data from the Danish Spine Database, not published). We know that patients are treated in different departments depending on the problem and the organization. Some patients are referred to one department, others to two or more, if needed.

In order to be able to monitor national and international guidelines for the management of patients with back pain, there is a call for a nationwide monitoring system for back pain disorders. The objective of such a system is to use information from the national registries to establish a national back pain registry monitoring the clinical course for patients with back pain. This will provide patient-level relevant and comprehensive information to the health care personnel enabling them to optimize the delivery of evidence-based care (The Danish Spine Database). The secondary goal is to include information on pain, disability, and quality of health, but this information is not available in the registries and has to be included by means of questionnaires.

In Denmark, all residents have free access to public health care, and all patients’ hospital services are collected centrally in national registries; it is thus possible to construct a database including information from different departments. The first step will be to develop a comprehensive database for patients with back pain using the Danish national central registries. The second step will be to assess the validity of the information given in the registry. However, patients seen in different departments are a challenge in register projects as the system xleaves no information on the linkage between visits related to the same clinical problem, and no information is given at the start and end of a course. Therefore, a model is needed for monitoring the management of patients with back pain, including details on the course across departments.

The aim of this study is to describe the interdisciplinary course of patients with back pain referred for diagnosing and treatment at a specialized spine center. This is a means to assess the face validity of the information given in the registry, using the information available in the Danish central registries.

This study is part of a large scale national study describing the course of back pain patients across sectors and specialties in the public health care system. To describe the individual course of the individual patient, algorithms are needed. This study describes the face validity of the individual course by information given in Danish central registries. The impact of such algorithms will provide the possibility to follow more than 50,000 incident patients seen in the hospital system each year.

## Methods

### Design and setting

This is a retrospective register based cohort study. Since 1968, all Danish residents have been assigned a unique civil registration number, which allows record linkage among different Danish medical and administrative registries on an individual level, and it is mandatory by law to report to the Danish health registries [11]. The Danish health care sector is tax financed and provides free coverage for all Danish citizens. Some services are based on co-payment (e.g. physiotherapy and chiropractor). The primary entry point to the Danish health care system is the General Practitioners who act as gatekeepers to specialized services.

#### Study population

The Region of Southern Denmark comprises 1.2 M inhabitants. All non-emergency patients with back pain from this region, who need specialized diagnostics and treatment, are referred to the Spine Center of Southern Denmark (SCSD). We used the Danish National Patient Registry (DNPR) to identify our study population, which includes all patients referred to the SCSD with a cervical as well as low back pain diagnosis [12] between 1 January 2011 and 31 December 2017. The DNPR contains all somatic hospital discharge diagnoses since 1977 and all outpatient diagnoses since 1995 as well as information on procedure codes for examinations and treatments [13]. The DNPR has been validated continuously and is considered a unique source for epidemiological research [13].

We chose the cohort from the SCSD because the authors have worked with this population clinically and for evaluation and have an in-depth knowledge of this group of patients, thus enabling assessment of face validity.

#### Definition of the interdisciplinary clinical course of back pain

We identified all back pain-related hospital visits and procedures using the DNPR including the following ICD10 classification codes: M41*, M503, M513, M53*, M54*, M62*, M998, M999. M43*, M45*, M46*, M47*, M480, M500–2, M510–2, M96*, M990–7. The information is stored in administrative courses linked to the hospitals’ departments. Patients who had contacts with more than one department during one clinical course will have more than one administrative course registered in the DNPR, which means that no information on the first and last visit in one continuous clinical course is registered in the DNPR. Therefore, to be able to follow the patient’s clinical course we have to define when a clinical course starts and ends using the information on the contacts during a certain time period, independently of the location for the contact. In our study, we defined the start of the patient’s first individual clinical course as the date of the first visit to the SCSD. The course ended when the patient had no contact during a specific time period after this date. Then a new individual clinical course can be initiated. In order to decide which time period without previous contact is most appropriate, we have constructed three models defined by the number of days that separate two individual clinical courses. In model I, we defined that a hospital contact with BPD starts with a new individual clinical course for the patient if the patient had no-hospital contact with BPD for at least 180 days, 365 days for model II, and 730 days for model III.

**Fig. 1:**
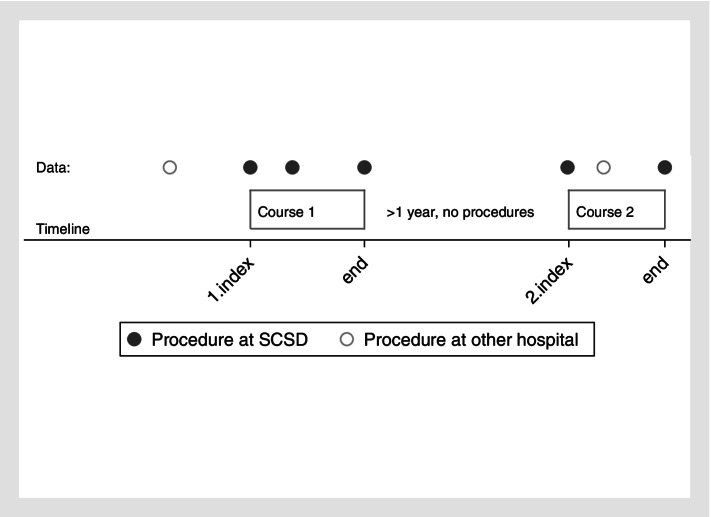
Example of clinical courses for a patient with back pain. Data includes procedures at the Spine Centre of Southern Denmark (SCSD) or in other hospital settings

We have described each population demographically to be able to choose the most appropriate model for further analyses. Figure 1 shows a detailed description of model II.

#### Variables used to describe the clinical back pain course

Each of the three constructed models led to a number of courses. We calculated the length of each course and the time between courses. We defined the treatment and investigation procedures at SCSD and other hospitals by procedure codes including codes for MRI, surgery, and rehabilitation. We coded the surgical procedures in the spine by specific SKS codes (KABC1, KABC20–21, KABC23, KABC26, KABC3–5, KNAG0, KNAG3–4, KNAG6, KNAG7, and KNAK4) [14]. Referral for rehabilitation in the municipality was coded as ZZ0175X. GP’s and chiropractors may refer patients directly for MRI before they are referred to the Spine Center. We identified the information on all diagnostic MRI-procedures by the SKS code UXME00–50. MRI scans performed up to 180 days before the index date or during the course [14] were included.

#### Definition of variables related to the clinical back pain course

We used the Danish National Health Service Register to obtain information on contacts to chiropractor or physiotherapist treatment 3 months before the index date. The Danish National Health Service Register does not contain information on diagnoses. We believe that chiropractor or physiotherapist treatment carried out a few days before (or after) hospital contact is probably related to the back pain episode. Chiropractor or physiotherapist treatment was included in the clinical course if the visit was registered from 90 days before index the date. According to the Danish guidelines a GP may refer a non-acute back pain patient to a specialized spine center after the patient has been in chiropractor or physiotherapist treatment for 3 months.

Moreover, we used the DNPR to define the following baseline variables: *Age* divided into two groups ≤60 and > 60, *Sex* (male or female), and somatic comorbidity – *CCI,* which was defined according to the Charlson comorbidity index (CCI) and classified into zero if CCI was equal to 0 and ≥ 1 if CCI was more than 0 [15, 16]. The CCI is an often used weighted comorbidity index derived from 19 different disorders; for each of the disorders a score between 0 and 6 depending on assumed severity is assigned. The CCI is calculated as the sum across these categories and ranges between 0 (with no diseases in the medical history qualifying for inclusion in the CCI) and 37 (a medical history representing all diseases of the highest severity, qualifying for inclusion in the CCI). To calculate the CCI we retrieved information on all 19 disorders for each patient up to 10 years before the index date, using the Danish National Patient Register.

### Statistical analysis

Three models describing the interdisciplinary course are established by information in the DNPR. We calculated differences according to treatment procedures and demographics in order to describe whether the models performed as expected. Since referral practice and hospital care guidelines were changed numerous times during the study period, we performed all descriptive analyses separately for three cohorts: 2011–2013, 2014–2015, and 2016–2017 to evaluate whether the different models were stable over time. All statistical analyses were conducted in STATA version 16 on Statistics Denmark’s research computers via a remote server.

## Results

In total, we identified 69,564 patients with at least one hospital contact with a back pain diagnosis from 1 January 2011 to 31 December 2017. Using model I, we identified 64,757 incident clinical back pain courses, 7842 patients (about 14%) had two clinical courses within the time period, 1474 had three or more, please refer to Table 1. The median length of the clinical course was 48 days, with 25 and 75% at 8 and 113 days, respectively. During the period 2011–2017, the median length of a clinical course was reduced from 60 days in the time period 2011–2013 to 43 days in the time period 2014–2015, and to 39 days in the time period 2016–2017. We defined 60,914 incident clinical back pain courses using model II. Nine percent of the study population or 4953 patients had two clinical courses within the time period, and less than 1 % had three or more clinical courses. The median length of the clinical course was 55 days with 25 and 75% at 13 and 143 days. As for model I the median length of a clinical course declines with time, from 70 days for the time period 2011–2013 to 43 days for the time period 2016–2017. Using model III, we identified 60,173 incident clinical back pain courses; only 5% of the patients had two or more clinical courses within the time period. The median length of the clinical course was 64 days with 25% and 75% at 18 and 211 days. The median length of a clinical course declined over time, from 83 days for the time period 2011–2013 to 49 days for the time period 2016–2017.

**Table 1:** Basic description of clinical courses by models

		Total	2011–2013	2014–2015	2016–2017
**Model I**:	**Number**	64,757	25,196	19,355	20,206
At least six months between courses	**Number of patients started # course during study period**				
	first	55,441 (85.6%)	23,638 (93.8%)	16,077 (83.1%)	15,726 (77.8%)
	second	7842 (12.1%)	1492 (5.9%)	2780 (14.4%)	3570 (17.7%)
	third or more	1474 (2.3%)	66 (0.3%)	498 (2.5%)	910 (4.5%)
	**Days of clinical course**				
	Median (25%;75%)	48 (8;113)	60 (15; 132)	43 (1; 107)	39 (1; 95)
	**Days between clinical course**				
	Median (25%;75%)	525 (308; 926)	324 (253; 489)	482 (301; 790)	728 (386; 1232)
**Model II:**	**Number**	60,914	23,763	18,085	19,066
At least one year between courses	**Number of patients started # course during study period**				
	first	55,031 (82.5%)	23,194 (97.6%)	16,101 (89.0%)	15,736 (82.5%)
	second	5433 (8.9%)	568 (2.3%)	1900 (10.5%)	2965 (15.6%)
	third or more	442 (0.7%)		84 (0.5%)	357 (1.9%)
	**Days of clinical course**				
	Median (25%;75%)	55 (13;143)	70 (22; 164)	49 (8; 134)	43 (3; 121)
	**Days between clinical course**				
	Median (25%;75%)	760 (526; 1139)	525 (434; 645)	681 (493; 953)	931 (602; 1381)
**Model III:**	**Number**	60,173	22,790	16,810	17,728
At least two years between courses	**Number of patients** **started # course during study period**				
	first	57,338 (95.1%)	22,718 (99.7%)	16,027 (95.3%)	15,758 (88.8%)
	second or more	2835 (4.9%)	72 (0.3%)	783 (4.7%)	1970 (11.2%)
	**Days of clinical course**				
	Median (25%;75%)	64 (18;211)	83 (28; 258)	58 (14; 203)	49 (8; 163)
	**Days between clinical course**				
	Median (25%;75%)	1108 (897; 1440)	812 (764; 895)	1009 (855; 1177)	1212 (958; 1548)

The distribution of performed clinical procedures is shown in Table 2. For model I, the proportion of clinical back pain courses where the patients received MRI scans declines over time from 86% (21,531 clinical courses) in the time period 2011–2013 to 83% (16,815 clinical courses) in the time period 2016–2017. In the entire study period, about 15% (16,815) of the clinical courses included surgical treatment, and about 30% (19,911 clinical courses) referral to rehabilitation in the municipal sector. The distribution of performed clinical procedures during the clinical back pain course was similar between models as well as changes in proportions of performed clinical procedures.

**Table 2:** Description of clinical procedures performed during the clinical course

		Total	2011–2013	2014–2015	2016–2017
**Model I:**	**MRI**				
At least six months between courses	Yes	54,062 (83.5%)	21,531 (85.5%)	15,716 (81.2%)	16,815 (83.2%)
	No	10,695 (16.5%)	3665 (14.5%)	3.639 (18.8%)	3391 (16.8%)
	**Surgical treatment**				
	Yes	9923 (15.3%)	3995 (15.9%)	2886 (14.9%)	3042 (15.1%)
	No	54,834 (84.7%)	21.201 (84.1%)	16,469 (85.1%)	17.164 (84.9%)
	**Referral to further rehabilitation**				
	Yes	19,911 (30.7%)	7055 (28.0%)	6180 (31.9%)	6676 (33.0%)
	No	41,846 (69.3%)	18,141 (72.0%)	13,175 (68.1%)	13,530 (67.0%)
**Model I I:**	**MRI**				
At least one year between courses	Yes	52,262 (85.8%)	20,899 (88.0%)	15,113 (83.6%)	16,250 (85.2%)
	No	8651 (14.2%)	2864 (12.0%)	2971 (16.4%)	2816 (14.8%)
	**Surgical treatment**				
	Yes	9944 (16.3%)	4022 (16.9%)	2871 (15.9%)	3051 (16.0%)
	No	50,969 (83.7%)	19,741 (83.1%)	15,213 (84.1%)	16,015 (84%)
	**Referral to further rehabilitation**				
	Yes	19,888 (32.7%)	7111 (29.9%)	6122 (33.9%)	6655 (34.9%)
	No	41,025 (67.3%)	16,652 (70.1%)	11,962 (66.1%)	12,411 (65.1%)
**Model III:**	**MRI**				
At least two years between courses	Yes	49,767 (86.8%)	20,284 (89.0%)	14,260 (84.8%)	15,223 (85.8%)
	No	7570 (13.2%)	2506 (11.0%)	2549 (15.2%)	2515 (14.2%)
	**Surgical treatment**				
	Yes	9854 (17.2%)	4115 (18.1%)	2850 (17.0%)	2889 (16.3%)
	No	47,483 (82.8%)	18,675 (81.9%)	13,959 (83.0%)	14,849 (83.7%)
	**Referral to further rehabilitation**				
	Yes	19,783 (34.5%)	7347 (32.2%)	6046 (36.0%)	6390 (36.0%)
	No	37,554 (65.5%)	15,443 (67.8%)	10,763 (64.0%)	11,348 (64.0%)

The patients’ baseline characteristics at the start of a clinical back pain course are available in Table 3. The distribution of all background variables was similar in the different cohorts. In all groups, about 50% of the patients were younger than 60 years, 55% were females. More than 70% of the study population had no comorbidity, and only 13% had multiple comorbidities. Approximately 40% of all the patients received chiropractic treatment or physiotherapy before the clinical back pain course. The patients’ baseline characteristics at the start of the clinical back pain course are similar for all three models.

**Table 3:** Baseline characteristics for all patients seen at the Spine Centre during seven years by information given in central registers^a^

		Total *N* = 60,913	2011–2013 *N* = 23,763	2014–2015 *N* = 18,084	2016–2017 *N* = 19,066
**Model I:**	**Age**				
At least six months between courses	Median (25%; 75%)	53 (42; 66)	51 (40; 64)	53 (42; 66)	54 (43; 67)
	**Sex**				
	Male	28,599 (44.2%)	11,057 (43.9%)	8538 (44.1%)	9004 (44.6%)
	Female	36,158 (55.8%)	14,139 (56.1%)	10,817 (55.9%)	11.202 (55.4%)
	**CCI**				
	0	46,615 (72%)	18.350 (73%)	13,864 (72%)	14,401 (71%)
	1	9587 (15%)	3665 (15%)	2913 (15%)	3009 (15%)
	> 1	8555 (13%)	3181 (13%)	2578 (13%)	2796 (14%)
	**Prior chiropractor or physiotherapist treatment**				
	Yes	25.711 (40%)	9567 (38%)	7764 (40%)	8380 (41%)
	No	39,046 (60%)	15,629 (62%)	11,591 (60%)	11,826 (59%)
**Model II:**	**Age**				
At least one year between courses	Median (25%; 75%)	52 (41; 65)	51 (40; 64)	53 (42; 66)	54 (43; 67)
	**Sex**				
	Male	27,016 (44.3%)	10,470 (44.1%)	8028 (44.4%)	8518 (44.7%)
	Female	33,897 (55.7%)	13,293 (55.9%)	10,056 (55.6%)	10.548 (55.3%)
	**CCI**				
	0	44,090 (72.4%)	17,410 (73.3%)	13,027 (72.0%)	13,653 (71.6%)
	1	8921 (14.7%)	3416 (14.4%)	2700 (14.9%)	2805 (14.7%)
	> 1	7902 (13.0%)	2937 (12.4%)	2357 (13.1%)	2608 (13.7%)
	**Prior chiropractor or physiotherapist treatment**				
	Yes	24,769 (40.7%)	9250 (38.9%)	7432 (41.1%)	8087 (42.4%)
	No	36,144 (59.3%)	14,513 (61.1%)	10,652 (58.9%)	10,979 (57.6%)
**Model III:**	**Age**				
At least two years between courses	Median (25%; 75%)	52 (41; 65)	51 (40; 64)	53 (41; 66)	54 (43; 67)
	**Sex**				
	Male	25,481 (44%)	10,019 (44%)	7493 (45%)	7969 (45%)
	Female	31,856 (56%)	12,771 (56%)	9316 (55%)	9769 (55%)
	**CCI**				
	0	42,024 (73%)	16,906 (74%)	12,267 (73%)	12,851 (72%)
	1	8194 (14%)	3202 (14%)	2546 (14%)	2546 (14%)
	> 1	7119 (12%)	2682 (12%)	2341 (13%)	2341 (13%)
	**Prior chiropractor or physiotherapist treatment**				
	Yes	23,709 (41%)	8986 (39%)	7063 (42%)	7660 (43%)
	No	33.628 (59%)	13,804 (61%)	9746 (58%)	10,078 (57%)

^a^Each patient is defined by the course of the patient

## Discussion

The aim of this study was, by use of information available in the central registers, to describe the face validity of a complex interdisciplinary clinical course for patients with back pain referred to a specialized spine center. We will use this model in future studies, evaluating the patients’ clinical course at different hospitals and departments. We proposed three models to define the index date and final date in a clinical back pain course by information given in DNPR. The models were applied in a cohort of patients who were referred to the SCSD with a back pain diagnosis from 1 January 2011 to 31 December 2017. We chose the SCSD as the population is well known to the authors and it is assumed that this knowledge will make it possible to assess the face validity of the model chosen to be used in future studies.

Most patients from the study population (> 80%) had only one clinical course during the study period which corresponds to the information from the local PRO-database, Spinedata [17] and to observations made by clinicians from the SCSD.

To define the period used when indicating the start of a new clinical course we used three definitions: 6 months, 1 year, and 2 years where the patient had no contact to the SCSD. The number of patients seen in the three periods is as expected reduced from model I to III, and the number of previous clinical courses are higher for model I than for model III. To check whether the days of the clinical course change over time, the median time is measured for each model for each of the three time periods 2011–2013, 2014–2015, and 2016–2017. For all models the number of days is reduced according to organizational changes, the trend is the same for all three models and we concluded that all the models provide this information. For all models, the number of days in between the clinical courses increases during time, which also is in accordance with organizational changes. The difference between the three models is the number of clinical courses for each of the patients. If the period with no contact before the index-date is too short too many patients will have more than one course, if it is too long the risk of being referred twice because of different symptoms is too high. For all three models, we found that significantly fewer second and third clinical back pain courses start in the time period 2011–2013 than in 2016–2017. This is consistent with our expectation as the study’s observation time begins in 2011. Since most patients from the study population had only one clinical course during the study period, the similarity of the baseline characteristics in all three models is not surprising.

The probability of starting the second and third clinical back pain courses in the period 2011–2013 is thus lower than starting the second and third clinical back pain courses in the period 2016–2017. This observation must be taken into consideration when identifying new cases by means of the central registers. From a clinical perspective model II is chosen as the most feasible and we concluded that future studies may include information on previous clinical courses and the diagnosis describing the cause of the visit to support the description of the population seen at a specialized spine center. Whether this assumption is valid in a national database, including the whole population of back pain patients, must be validated in the national database.

To support the decision on using model II, the population in each of the three models was described by procedures performed during the clinical course and by baseline characteristics available in the registers. The MRI-rate declines a little through the time periods for all models. The surgical rate is stable and the referral-rate for rehabilitation increases for all models. Characteristics available in the registers were thereafter reported: age, sex, CCI, and prior treatment is stable in between the models, and changes during time is the same. It supports the conclusion that the models are rather stable when we look at demographics and treatment.

### Strengths

The strengths of this method using central registers are multiple. All patients seen at defined locations will be included in the cohort, and it is possible to follow the patients treated at more than one department and hospital after the first visit. It is possible to include information on diagnostic procedures as well as treatment on the actual clinical course. The method can be used nationally while all departments report the same data on dates and procedures into the registers.

### Limitations

The introduced model has some weaknesses. The validity of our model depends on the validity of the DNPR. Although it has been shown that DNPR has very high completeness according to the registration of clinical visits [17–19], to our knowledge there are no studies explicitly validating the registration of back pain diagnosis codes. Still, the misclassification for the current purpose will be limited. As a limitation, we could also mention a possible misclassification as patients seen at the spine center repetitively with more than 1 year between contacts has two different clinical courses. To deal with this, the previous contact will be included in future analyses. Another limitation is that diagnoses are not included leaving the possibility that the patients have lower back pain as well as neck pain. Because of limited time between visits, they will be counted as one course with one diagnosis defined by the latest. This probably only involves a minority, and a secondary diagnosis must be taken into consideration in future analyses.

The described distribution of the patients’ baseline characteristics, as well as the clinical procedures, corresponds to what we observed on an everyday basis in the clinic [9]. We found that there are about 9000 clinical back pain courses per year corresponding to the daily clinical practice information.

The access to information registered in the DNPR leaves the opportunity to describe the population by sociodemographic information as the present. It also leaves additional opportunities for research and clinical control in the area of back pain disorders. Clinical back pain courses can be linked to other Danish central registries such as the Danish Prescription Registry [20–22], The Danish National Health Service Register [23] or the Danish Register for Evaluation of Marginalisation [24] and provide the chance to investigate the pattern of the patients’ medicine use and workability.

Our model for generating interdisciplinary clinical back pain courses on patients referred to the SCSD can be applied to all patients with back pain in Denmark, leaving the possibility to compare back pain treatment quality across departments and hospitals in the country. A reduction in our algorithm’s broad usage could be differences in the classification of diagnoses across hospitals. The SCSD is a specialized spine center and thus more likely to use a standardized coding practice according to the ICD-10 classification of disease since the coding practice could be different; usage of the introduced model needs to be tested before applying it nationally.

Patients solely seen in the primary sector are registered by diagnosis, therefore it is not possible to identify the first visit for patients seen by the GP, physiotherapist, or chiropractor. More than two thirds of the patients seen by health professionals are seen in the primary sector. The clinical course described in this paper is representative for the patients referred to a spine center, which has to be taken into consideration when register data is used to describe the clinical course for back pain patients. When patients seen in the primary sector are registered by diagnosis, the model described can be used for all patients seen by health professionals.

## Conclusions

The back pain course for the population of patients with back pain, referred for diagnosis and treatment at a specialized regional spine center, can be identified using the Danish central registers. The start and end of an interdisciplinary clinical course can be defined using time-intervals between clinical contacts. The one-year time interval fits best the clinicians’ everyday observations. Other clinically important information, such as intensity of pain, disability, and quality of life can be added to the back pain course by questionnaires, if needed.

## Data Availability

The data that support the findings of this study was accessed remotely on a secure platform at Statistics Denmark. Any access to data requires permission from Statistics Denmark and the Danish Cancer Society: Danish Cancer Society Research Center, Strandboulevarden 49, 2100 Copenhagen, Denmark.

## Abbreviations

SCSD: Spine Center of Southern Denmark
DNPR: Danish National Patient Registry
BPD: Back pain diagnosis
ICD10: International Classification of Diseases and Related Health Problems version 10
MRI: Magnetic Resonance Imaging
CCI: Charlson comorbidity index
GP: General practitioner
